# Association Between Survival and Metastatic Site in Mismatch Repair–Deficient Metastatic Colorectal Cancer Treated With First-line Pembrolizumab

**DOI:** 10.1001/jamanetworkopen.2023.0400

**Published:** 2023-02-22

**Authors:** Bahar Saberzadeh-Ardestani, Jeremy C. Jones, Joleen M. Hubbard, Robert R. McWilliams, Thorvardur R. Halfdanarson, Qian Shi, Mohamad Bassam Sonbol, Jonathan Ticku, Zhaohui Jin, Frank A. Sinicrope

**Affiliations:** 1 Gastrointestinal Research Unit, Mayo Clinic, Rochester, Minnesota; 2 Department of Oncology, Mayo Clinic, Jacksonville, Florida; 3 Department of Oncology, Mayo Clinic, Rochester, Minnesota; 4 Department of Quantitative Health Science, Mayo Clinic, Rochester, Minnesota; 5 Department of Oncology, Mayo Clinic, Phoenix, Arizona; 6 Mayo Clinic Health System, La Crosse, Wisconsin

## Abstract

**Importance:**

Metastatic colorectal cancer (mCRC) with deficient DNA mismatch repair (dMMR) shows frequent and durable responses to programmed cell death 1 blockade. While most of these tumors are sporadic and observed in older patients, first-line pembrolizumab data are limited to findings from the KEYNOTE-177 trial (A Phase III Study of Pembrolizumab [MK-3475] vs Chemotherapy in Microsatellite Instability-High [MSI-H] or Mismatch Repair Deficient [dMMR] Stage IV Colorectal Carcinoma).

**Objective:**

To investigate outcome with first-line pembrolizumab monotherapy in mostly older patients with dMMR mCRC at a multisite clinical practice.

**Design, Setting, and Participants:**

This cohort study included consecutive patients with dMMR mCRC who received pembrolizumab monotherapy between April 1, 2015, and January 1, 2022, at Mayo Clinic sites and the Mayo Clinic Health System. Patients were identified from review of electronic health records at the sites, which included the evaluation of digitized radiologic imaging studies.

**Intervention:**

Patients with dMMR mCRC received first-line pembrolizumab, 200 mg, every 3 weeks.

**Main Outcomes and Measures:**

The primary study end point was progression-free survival (PFS), which was analyzed using the Kaplan-Meier method and a multivariable stepwise Cox proportional hazards regression model. Clinicopathological features, including metastatic site and molecular data (*BRAF* V600E and *KRAS*), were also analyzed along with tumor response rate, which was determined using Response Evaluation Criteria in Solid Tumors, version 1.1.

**Results:**

The study cohort included 41 patients (median [IQR] age at treatment initiation, 81 [76-86] years; 29 females [71%]) with dMMR mCRC. Of these patients, 30 (79%) had the *BRAF* V600E variant and 32 (80%) were classified as having sporadic tumors. Median (range) follow-up was 23 (3-89) months. Median (IQR) number of treatment cycles was 9 (4-20). Overall response rate was 49% (20 of 41 patients), including 13 patients (32%) with complete responses and 7 (17%) with partial responses. Median (IQR) PFS was 21 (95% CI, 6-39) months. Liver as a site of metastasis was associated with significantly poorer PFS vs nonliver metastasis (adjusted hazard ratio, 3.40; 95% CI, 1.27-9.13; adjusted *P* = .01). Complete and partial responses were observed in 3 patients (21%) with liver metastasis vs 17 patients (63%) with nonliver metastases. Treatment-related grade 3 or 4 adverse events were observed in 8 patients (20%), 2 of whom discontinued therapy; there was 1 treatment-related death.

**Conclusions and Relevance:**

This cohort study found a clinically significant prolongation of survival in older patients with dMMR mCRC who were treated with first-line pembrolizumab in routine clinical practice. Furthermore, liver vs nonliver metastasis was associated with poorer survival in this patient population, which suggests that the metastatic site has implications for survival outcome.

## Key Points

**Question:**

What is the association between survival and metastatic site in patients with mismatch repair–deficient metastatic colorectal cancer who were treated with pembrolizumab as a first-line therapy?

**Findings:**

In this cohort study of 41 patients with mismatch repair–deficient metastatic colorectal cancer who received first-line pembrolizumab, liver vs nonliver metastasis was associated with significantly poorer progression-free survival.

**Meaning:**

The finding that liver vs nonliver metastasis was associated with decreased survival in this patient population suggests that metastatic site may have implications for treatment and survival outcome.

## Teaser

This cohort study assesses the clinicopathological features, outcomes, and adverse events of older patients with metastatic colorectal adenocarcinomas.

## Introduction

Colorectal cancer (CRC) with deficient DNA mismatch repair (dMMR) or high microsatellite instability (MSI-H) is characterized by high tumor mutation burden that typically trigger an antitumor immune response in the tumor microenvironment.^1,2,3,4^ Approximately 15% of CRC cases have dMMR, which is more commonly of sporadic origin due to methylation of the *MLH1* gene promoter or is hereditary due to a germline sequence variant in an *MMR* gene (*MLH1*, *MSH2*, *MSH6*, and *PMS2*) that is associated with Lynch syndrome.^5^ Sporadic CRC with dMMR is enriched with *BRAF* V600E variants and is more common in older patients.^6,7^ The observed better clinical outcome of patients with dMMR vs proficient MMR colon cancer has been shown to be attenuated with advancing tumor stage, and poorer outcome is reported in dMMR metastatic CRC (mCRC) that is not treated with immunotherapy.^8,9^

Programmed cell death 1 (PD-1) blockade with pembrolizumab or nivolumab has produced frequent and durable responses in a substantial proportion of patients with dMMR mCRC, which led to the US Food and Drug Administration approvals of these antibodies for patients whose tumors have progressed after treatment with fluoropyrimidine, oxaliplatin, and irinotecan.^10,11,12,13^ The phase 3 open-label KEYNOTE-177 trial (A Phase III Study of Pembrolizumab [MK-3475] vs Chemotherapy in Microsatellite Instability-High [MSI-H] or Mismatch Repair Deficient [dMMR] Stage IV Colorectal Carcinoma) demonstrated that first-line pembrolizumab was superior to chemotherapy (fluorouracil-based therapy with or without bevacizumab or cetuximab) with respect to progression-free survival (PFS) with fewer treatment-related adverse events (AEs).^14^ Results of the KEYNOTE-177 trial led to Food and Drug Administration approval of pembrolizumab monotherapy for previously untreated patients with dMMR unresectable or metastatic CRC.^15^ However, the 44% overall response rate (ORR) of pembrolizumab in the trial underscores the frequent presence of intrinsic or acquired treatment resistance; accordingly, the need for predictive biomarkers for response to anti–PD-1 antibodies remains unmet.^16^

More than 60% of new cancer cases and more than 70% of cancer-related deaths are observed in older adults,^17^ whose numbers are steadily rising worldwide.^18^ While the majority of dMMR CRC tumors are sporadic and are observed in older patients, published data for pembrolizumab as a first-line therapy for this tumor subset are limited to those in a single clinical trial, whose cohort had inherent biases of younger age and favorable performance status.^19^ Older patients with cancer have often been undertreated and are underrepresented in clinical trial cohorts compared with younger patients.^20,21,22^ In the present study, we aimed to investigate outcome with first-line pembrolizumab monotherapy in mostly older patients with dMMR mCRC at a multisite clinical practice.

## Methods

### Study Cohort

This multicenter retrospective cohort study included consecutive patients with dMMR or MSI-H metastatic colorectal adenocarcinomas who received pembrolizumab as a first-line therapy between April 1, 2015, and January 1, 2022, at the Mayo Clinic 3-sites Comprehensive Cancer Center (n = 33) and at the Mayo Clinic Health System (n = 8). These patients were identified from the electronic health record (EHR) system at the sites using Epic Slicer Dicer (Epic Systems Corporation). Review of the EHRs included the evaluation of digitized radiologic imaging studies. This study was approved by the Mayo Clinic Institutional Review Board, which waived the informed consent requirement because of the study’s retrospective minimal-risk nature. We followed the Strengthening the Reporting of Observational Studies in Epidemiology (STROBE) reporting guideline.

Study variables included patient age at the start of immunotherapy (years), sex (female vs male), Eastern Cooperative Oncology Group Performance Status (PS) score (0-1 vs ≥2),^23^ metastatic disease at presentation (metachronous vs synchronous), primary tumor location (right colon, left colon, or both [synchronous]), carcinoembryonic antigen (CEA), site of tumor metastasis, and number of involved metastatic sites. Data for treatment-related AEs and their grade were also abstracted according to the Common Terminology Criteria for Adverse Events, version 5.0 (National Cancer Institute). Pembrolizumab, 200 mg, was administered every 3 weeks to patients. Primary tumors had been analyzed for DNA MMR proteins by immunohistochemistry, for MSI by polymerase chain reaction, or for somatic variant profiling by next-generation sequencing. Molecular data included *KRAS* (wild type vs variant) and *BRAF* V600E variant (present vs absent). Tumor mutation burden was available in those patients with next-generation sequencing data. Tumors with loss of MLH1 protein or both MLH1 and PMS2 proteins were categorized as sporadic if they harbored the *BRAF* V600E variant^24^ and/or had *MLH1* gene promoter methylation analyzed by bisulfite sequencing.^25^ Patients were categorized as having Lynch syndrome if their tumors showed loss of MSH2 protein and/or MSH6 or PMS2 protein alone or had a germline sequence variation in any *MMR* gene.

The dates of first and last dose of pembrolizumab and the number of cycles received were recorded. The date of best response and the date of last follow-up with determination of disease status were abstracted from the EHR. Tumor response to pembrolizumab was determined according to the Response Evaluation Criteria in Solid Tumors, version 1.1 (RECIST 1.1) and categorized as follows: complete response, partial response, stable disease, and progressive disease.

### Statistical Analysis

The primary study end point was PFS, which encompassed the time from first dose of pembrolizumab to first evidence of disease progression or death from any cause, whichever occurred first. Patients who discontinued treatment for reasons other than progressive disease were followed up for disease status until disease progression, initiation of another cancer treatment, or loss to follow-up. Overall survival was calculated from the date of first dose of pembrolizumab to the date of death from any cause, and living patients were censored at last follow-up when the patient was known to be alive regardless of disease status. The ORR was calculated as the proportion of patients who had a complete or partial response to therapy; disease control rate was calculated as the proportion of patients with a complete response, partial response, or stable disease. Among patients with a complete response or partial response, the duration of response was defined as the time from best response to first disease progression or death from any cause, whichever occurred first, and patients were censored at the date of last follow-up. All analyses were based on locking of the study database on July 18, 2022.

Continuous variables were described as median (IQR) values. Fisher exact test was used to assess the association between 2 categorical variables. Kaplan-Meier method and Cox proportional hazards regression models were used to analyze PFS by clinicopathological variables. Multivariable analyses of study variables were evaluated by stepwise Cox proportional hazards regression modeling.^26^ Adjusted hazard ratios (aHR) and 95% CIs were reported. Two-sided *P* < .05 indicated the statistical significance of findings for all analyses. Statistical analyses were performed with R, version 4.2.0 (R Foundation for Statistical Computing).

## Results

### Patient and Clinicopathological Characteristics

The cohort consisted of 41 consecutive patients with dMMR or MSI-H mCRC who received pembrolizumab as a first-line therapy. Clinicopathological characteristics of patients are shown in the Table. Patients were mostly older (80.5% were aged ≥65 years), with a median (IQR) age at treatment initiation of 81 (76-86) years, and the cohort included 29 females (71%) and 12 males (29%). Eight patients (22%) had a PS score of 2 or higher.

**Table.** Clinicopathological Characteristics, Outcomes, and Adverse Events of Patients With Mismatch Repair–Deficient Metastatic Colorectal Cancer

Variable^a^^,^^b^	No. (%)
Total No. of patients	41
Age at treatment initiation, median (IQR), y	81 (76-86)
Sex	
Female	29 (71)
Male	12 (29)
Metastatic disease at presentation	
Metachronous	12 (29)
Synchronous	29 (71)
No. of metastatic site	
1	14 (34)
2	13 (32)
≥3	14 (34)
Peritoneum metastasis	
Yes	19 (46)
No	22 (54)
Liver metastasis	
Yes	14 (34)
No	27 (66)
Lung metastasis	
Yes	12 (29)
No	29 (71)
Primary tumor location	
Right colon	30 (74)
Left colon	10 (24)
Both(synchronous)	1 (2)
ECOG Performance Status score	
0-1	29 (78)
≥2	8 (22)
*KRAS*	
Variant	2 (5)
Wild type	35 (95)
*BRAF* V600E variant	
Present	30 (79)
Absent	8 (21)
Lynch syndrome	
No	32 (80)
Yes	8 (20)
TMB, median (IQR), mut/Mb^c^	51 (37-73)
CEA, median (IQR), ng/mL	6 (3-15)
Progression-free survival	
No. of events	23
Median (95% CI), mo	21 (6-39)
Overall survival	
No. of events	15
Median (95% CI), mo	36 (14-NR)
Tumor response per RECIST 1.1	
Complete response	13 (32)
Partial response	7 (17)
Stable disease	3 (7)
Disease progression	15 (37)
ORR, %	49
DCR, %	56
At 12 mo	46
At 24 mo	24
At 36 mo	12
Duration of response, median (95% CI), mo	42 (9-NR)
No. of AEs	
1	16 (39)
2	3 (7)
3	1 (2)
Toxic effect	
Dermatitis	4 (10)
Kidney injury	3 (7)
Pneumonitis	3 (7)
Colitis	2 (5)
Arthritis	2 (5)
Hepatitis	2 (5)
Hypothyroidism	2 (5)
Adrenal insufficiency	1 (2)
Myocarditis	1 (2)
Toxic effect grade	
1-2	11 (27)
3-4	8 (20)
Led to treatment discontinuation	2 (5)
5	1 (2)

Abbreviations: AEs, adverse events; CEA, carcinoembryonic antigen; DCR, disease control rate; ECOG, Eastern Cooperative Oncology Group; mut/Mb, mutation per megabase; NR, not reached (the upper limit of the survival curve has not reached 50%); ORR, overall response rate; RECIST 1.1, Response Evaluation Criteria in Solid Tumors, version 1.1; TMB, tumor mutation burden.

SI conversion factor: To convert CEA to micrograms per liter, multiply by 1.0.

^a^ Missing values were not included in percentages.

^b^ Sum of numbers from each subcategory may not equal the total reported cases due to missing values.

^c^ TMB was available for 14 patients.

The median (range) follow-up was 23 (3-89) months, and the median (IQR) number of treatment cycles received was 9 (4-20). The most common site of metastatic disease was nonregional lymph nodes (25 [61%]), followed by peritoneum (19 [46%]), liver (14 [34%]), and lung (12 [29%]). The median (IQR) number of sites of metastasis was 2 (1-5) in patients with liver metastasis compared with 1 (1-3) in patients with nonliver metastasis (*P* = .01). The *BRAF* V600E variant was present in 30 tumors (79%). Loss of both *MLH1* and *PMS2* expression was observed in 32 patients (86%), loss of *MSH6* expression in 4 patients (11%), and loss of *MLH1* expression alone in 1 patient (3%). Patients were classified as having sporadic (32 [80%]) or hereditary (Lynch syndrome–related) (8 [20%]) cancers. Most patients (30 [73%]) had right-sided primary tumors (Table).

### Patient Response and Survival

**Figure 1.**
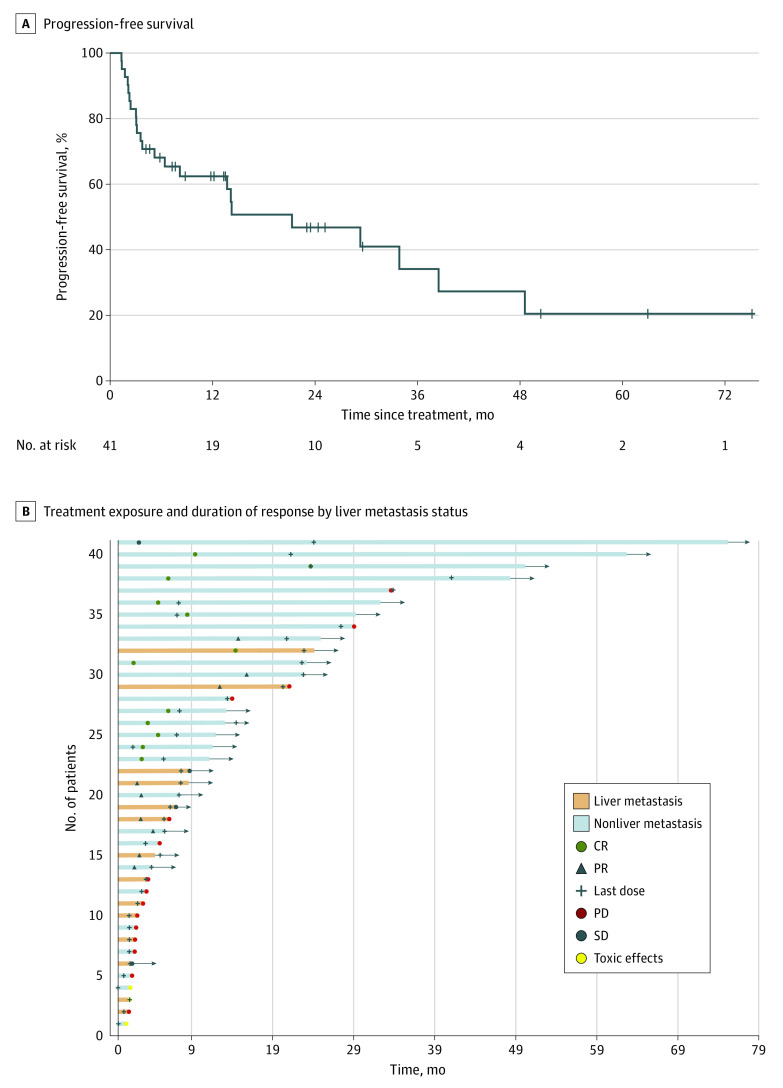
Outcome of Patients With Mismatch Repair–Deficient Metastatic Colorectal Cancer Treated With Pembrolizumab as First-line Therapy CR indicates complete response; PD, progressive disease; PR, partial response; and SD, stable disease. The arrows represent continued treatment.

Among 41 patients, median PFS was 21 (95% CI, 6-39) months (Figure 1A). Objective response (complete response and partial response) was observed in 20 patients (for an ORR of 49%), and disease control (complete response, partial response, and stable disease) was achieved in 23 patients (56%). There were 13 patients (32%) with complete responses, 7 (17%) with partial responses, 3 (7%) with stable disease, and 15 (37%) with progressive disease (Table). Thirty-eight patients were evaluated for response since 3 discontinued pembrolizumab due to toxic effects. Nineteen patients (46%) were found to have disease control at 12 months, 10 (24%) at 24 months, and 5 (12%) at 36 months of clinical follow-up. Five of 13 patients (39%) who achieved complete response remained free of disease for 3 or more years at median follow-up. Analysis of overall survival revealed a median value of 36 months (95% CI, 14 months to not reached [the upper limit of the survival curve has not reached 50%]). Median duration of response was 42 months (95% CI, 9 months to not reached).

### Response Rate by Site of Metastasis

**Figure 2.**
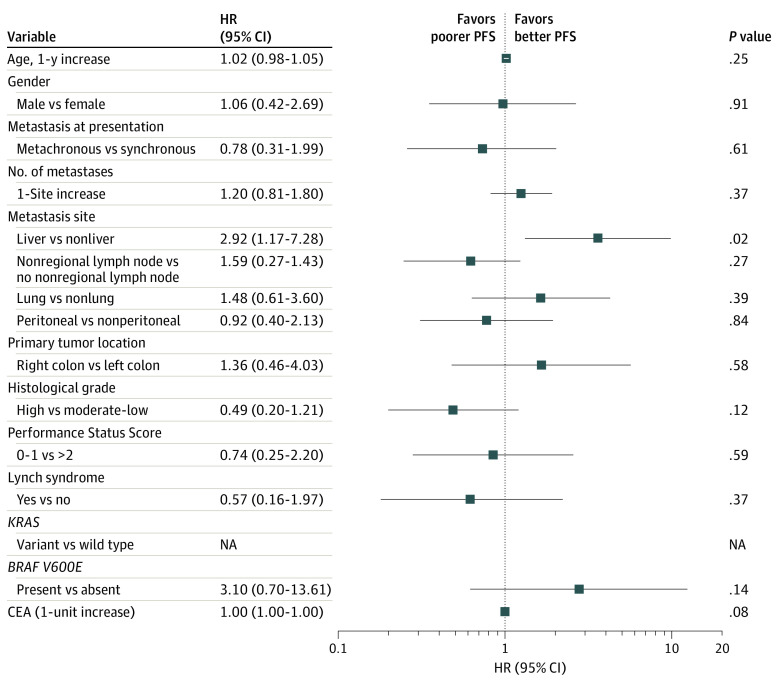
Bivariable Analysis of Tumor-Related Variables Associated With Progression-Free Survival (PFS) in Patients With Mismatch Repair–Deficient Metastatic Colorectal Cancer CEA indicates carcinoembryonic antigen; HR, hazard ratio; NA, not applicable.

In the cohort, 14 patients (34%) had liver metastasis and 1 of them had the liver as the only site of metastatic disease (Table). Bivariable analysis showed that metastasis to the liver, but not other sites, was associated with poorer PFS in those who received pembrolizumab (HR, 2.92; 95% CI, 1.17-7.28; *P* = .02) (Figure 2). At the time of diagnosis of metastatic disease, the median (IQR) sum of the largest diameter of each liver metastasis was 1.9 (1.1-3.2) cm, and the median (IQR) number of liver metastases was 2 (1-2). The median (IQR) pretreatment serum CEA level was similar between patients with liver and patients with nonliver metastasis (5.3 [2.9-8.9] ng/mL vs 6.3 [2.4-15.8] ng/mL; *P* = .88; to convert to micrograms per liter, multiply by 1.0). The PS score was also similar among these 2 groups. Complete and partial responses to pembrolizumab were observed in 3 patients (21%) with liver metastasis compared with 17 patients (63%) with nonliver metastatic disease (Figure 1B). The rate of lung metastasis was 43% (6 of 14) in patients with liver metastasis compared with 22% (6 of 27) in those with nonliver metastasis; peritoneal disease was also similar among these groups.

**Figure 3.**
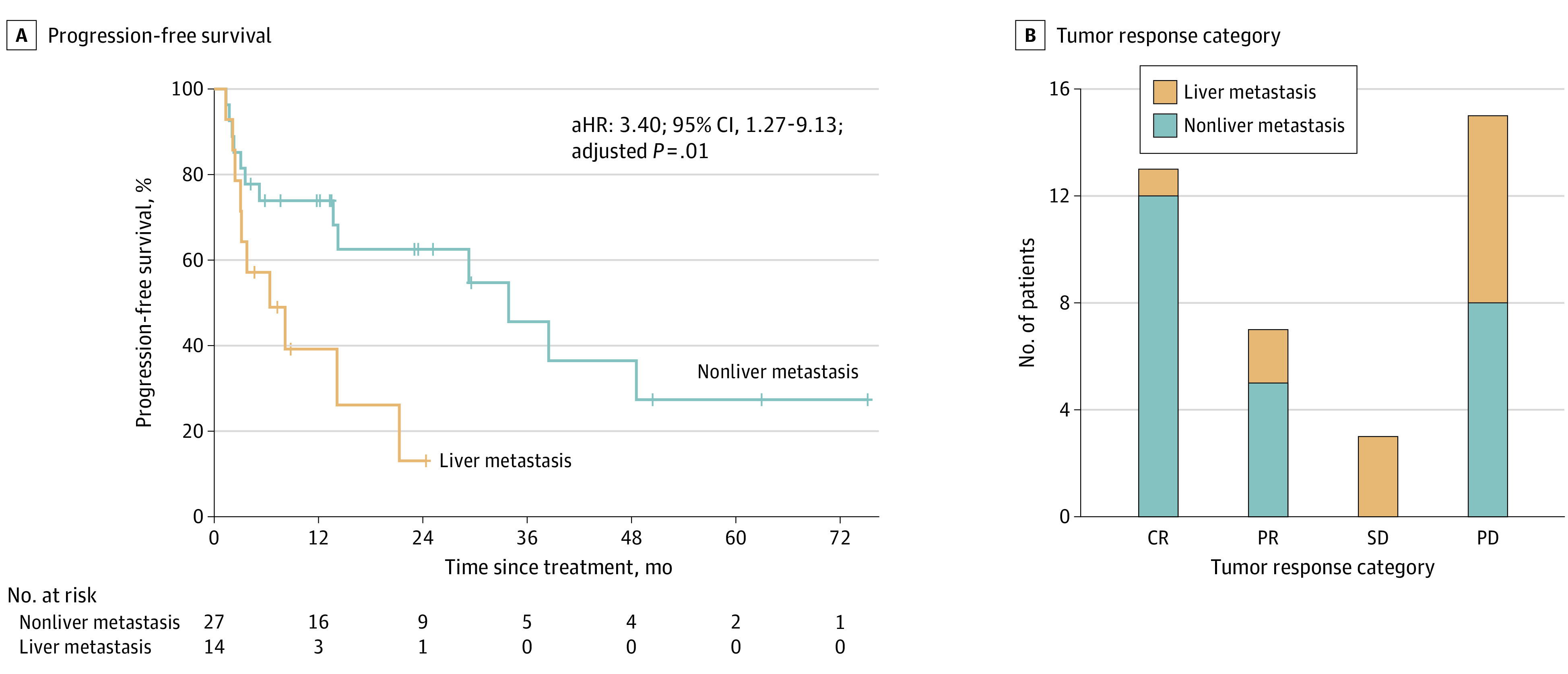
Outcome by Liver Metastasis in Patients With Mismatch Repair–Deficient Metastatic Colorectal Cancer Treated With Pembrolizumab as First-line Therapy CR indicates complete response; PD, progressive disease; PR, partial response; SD, stable disease.

Liver vs nonliver metastasis was associated with significantly poorer PFS in patients who were treated with pembrolizumab (Figure 3A). The median PFS of patients with liver metastasis was 6 (95% CI, 2-21) months compared with 34 months (95% CI, 14 months to not reached) for those with nonliver metastasis. Furthermore, the disease control rate at 12 months was 20% (3 of 14) in patients with liver metastasis compared with 59% (16 of 27) in those with nonliver metastasis (*P* = .04). Liver vs nonliver metastasis was associated with tumor response: 1 patient with liver metastasis had complete response (7.7%), 2 had partial response (28.6%), and 7 had progressive disease (46.7%) (Figure 3B). Among patients with a complete response, liver metastasis was present in 1 of 13 patients (8%), whereas liver metastasis was found in 7 of 15 patients (47%) who experienced disease progression. The association of liver metastasis with poorer PFS was maintained when the cohort was limited to patients older than 70 years who received pembrolizumab (HR, 3.37; 95% CI, 1.22-9.33; *P* = .02).

Next, we performed forward stepwise Cox proportional hazards regression modeling of study variables and found that the liver as the site of metastasis and *BRAF* sequence variation were the only variables that remained in the multivariable analysis (*BRAF* V600E: aHR, 3.14; 95% CI, 0.71-13.87; adjusted *P* = .13). Of these 2 variables, only liver metastasis was associated with poorer PFS after adjustment for other covariates (aHR, 3.40; 95% CI, 1.27-9.13; adjusted *P* = .01). When the number of sites of metastatic disease and CEA were added to the multivariable model, liver metastasis remained associated with poorer PFS after adjustment for these covariates (aHR, 3.01; 95% CI, 1.05-8.67; adjusted *P* = .04).

### Adverse Events

In the overall cohort, 20 patients (49%) experienced a treatment-related AE, regardless of grade. Eight patients (20%) experienced a grade 3 or 4 AE (2 had pneumonitis, 1 had adrenal insufficiency, 2 had kidney failure, 1 had thyrotoxicosis with subsequent hypothyroidism, 1 had colitis, and 1 had hepatitis) (Table), which led to 2 (5%) discontinuing treatment due to toxic effects (eg, kidney failure and pneumonitis) attributed to pembrolizumab. One death (grade 5 toxic effect) was attributed to treatment-related myocarditis (Table).

## Discussion

Among 41 patients with sporadic dMMR tumors, treatment with pembrolizumab resulted in a median PFS of 21 months and an ORR of 49% at a median follow-up of 23 months. These results indicated a clinically meaningful long-term benefit of pembrolizumab; specifically, 39% of patients who achieved complete response remained free of disease for 3 or more years at median follow-up, which is likely to represent cure of their metastatic disease. In the KEYNOTE-177 trial, which randomized patients with dMMR mCRC to either first-line pembrolizumab or chemotherapy, 47.7% of patients were 65 years or older compared with 80.5% in the present study. While the PS score of patients in the KEYNOTE-177 trial was limited to 0 to 1, the present cohort included 8 patients (22%) who had PS score of 2 or 3; despite this finding and the more advanced age of the patients in this study, PFS of 21 months was at least as favorable as the median PFS of 16.5 months reported in the KEYNOTE-177 trial.^14,27,28^

Advanced patient age has been shown to be associated with better response to cancer immunotherapy,^29,30,31,32^ which was reported in a meta-analysis of 19 randomized clinical trials that included 11 157 patients, the majority of whom had advanced melanoma or non–small cell lung cancer.^33^ However, the association of age and response to an immune checkpoint inhibitor (ICI) among patients with mCRC is controversial. Among 538 patients with advanced melanoma who were treated across 8 institutions worldwide, those over age 60 years had a better response to pembrolizumab than younger patients, with the odds of disease progression decreasing 13% for every decade of patient age at treatment initiation.^34^ A potential contributory mechanism is suggested by data from primary and metastatic melanoma tissues that showed older vs younger patients had an increased ratio of effector CD8^+^ to immune suppressive regulatory T cells.^34^ Similarly, a higher number of CD8^+^ T cells and a lower level of FOXP3^+^ (forkhead box P3) regulatory T cells were observed among melanomas in older vs younger mice.^34^

In patients with mCRC who underwent systemic chemotherapy, those with liver-only metastases vs all other sites of metastases had similar median overall survival rates, as shown in a multivariable analysis of the ARCAD (Analysis and Research in Cancers of the Digestive System) database.^35^ In the present study cohort, 34% of patients with dMMR mCRC had liver metastases, and we made the novel observation that metastatic disease that included the liver (vs nonliver metastases) was associated with significantly poorer PFS for patients who received pembrolizumab; this finding was independent of covariates and the number of sites of synchronous metastatic disease or CEA as a measure of tumor burden. A relevant observation was that ICI treatment in patients with proficient MMR mCRC and liver metastases was associated with significantly poorer PFS compared with patients with nonliver metastases.^36^ Specifically, patients with proficient MMR microsatellite-stable mCRC who were treated with nivolumab and regorafenib had substantially reduced response rates and shorter survival than those with liver vs nonliver metastases.^37,38^ Resistance to ICIs in patients with liver metastases has also been reported in other cancer types, including melanoma, urothelial and kidney carcinomas, and non–small cell lung cancer.^39^ A potential mechanism contributing to this observation may be liver-promoted, systemic immune tolerance.^40^ In preclinical models, liver metastasis was associated with reduced effectiveness of immunotherapy due to macrophage-mediated, intratumoral T-cell elimination.^39,41^ Furthermore, data from preclinical models of liver metastases showed that siphoning of activated CD8^+^ T cells from the systemic circulation by the liver played a role in immunotherapy resistance.^39^ Entrapment of cytotoxic T cells may prevent their expansion and activation. Further study of the mechanism underlying poorer survival of patients with liver metastasis is needed, including evaluation of novel therapeutic strategies to improve clinical outcome in this common setting.

The study cohort was highly enriched with sporadic vs Lynch syndrome–related dMMR mCRC, reflecting that approximately two-thirds of dMMR tumors at diagnosis were sporadic.^5^ Consistent with the demographic characteristics of the study cohort, sporadic dMMR tumors were associated with older age at diagnosis and female predominance.^5,42^ Sporadic dMMR tumors were enriched with the *BRAF* V600E point variant, which was detected in 79% of tumors compared with 37.0% in the KEYNOTE-177 trial.^14^ However, no difference in PFS was found based on the mechanism of MMR deficiency or primary tumor sidedness in the present study or in other reports of patients who were treated with pembrolizumab.^13,43,44^ The presence of the *BRAF* V600E variant was not associated with survival in the multivariable model, and in prior studies^12,13,43,44^ in dMMR mCRC, neither *BRAF* V600E variant, *KRAS* variant, nor the level of programmed death ligand 1 protein expression was able to distinguish responders from nonresponders to ICI therapy.

The safety profile of pembrolizumab monotherapy in the present study was consistent with that in the KEYNOTE-177 trial^14,27^ despite the more advanced age and poorer PS score of patients in this cohort.

### Strengths and Limitations

Strengths of this study were inclusion of consecutive patients from a multisite clinical practice, where imaging studies were reviewed, and use of RECIST 1.1 to determine response. Study limitations included the retrospective nature of the study and the relatively modest size of the cohort. Furthermore, the results, based on model selection, should be interpreted with cautious skepticism. Moreover, we could not directly examine the effectiveness of pembrolizumab among patients with liver vs nonliver metastasis since we did not have a control group that did not receive pembrolizumab.

## Conclusions

In this cohort study of patients with dMMR mCRC, we found that first-line therapy with pembrolizumab was a highly effective treatment in routine clinical practice despite the advanced age of the patients. The observation that a patient subset was free of disease after 3 or more years of follow-up suggests the likelihood of potential cure of metastatic disease. The finding that liver vs nonliver metastasis was associated with poorer survival in patients who received pembrolizumab suggests that metastatic site has implications for survival outcome and treatment. Despite the advanced age of the patient population, the toxic effects were manageable and similar to the AEs observed in the younger patient population of the KEYNOTE-177 trial. Findings of this study indicate that age alone should not exclude patients with dMMR mCRC from receiving ICI treatment.

## Notes

Supplement.
Data Sharing Statement

## References

[1] Muzny DM, Bainbridge MN, Chang K, ; Cancer Genome Atlas Network. Comprehensive molecular characterization of human colon and rectal cancer. Nature. 2012;487(7407):330-337. doi:10.1038/nature11252 22810696 PMC3401966

[2] Schwitalle Y, Kloor M, Eiermann S, . Immune response against frameshift-induced neopeptides in HNPCC patients and healthy HNPCC mutation carriers. Gastroenterology. 2008;134(4):988-997. doi:10.1053/j.gastro.2008.01.015 18395080

[3] Kim H, Jen J, Vogelstein B, Hamilton SR. Clinical and pathological characteristics of sporadic colorectal carcinomas with DNA replication errors in microsatellite sequences. Am J Pathol. 1994;145(1):148-156. 8030745 PMC1887287

[4] Llosa NJ, Cruise M, Tam A, . The vigorous immune microenvironment of microsatellite instable colon cancer is balanced by multiple counter-inhibitory checkpoints. Cancer Discov. 2015;5(1):43-51. doi:10.1158/2159-8290.CD-14-0863 25358689 PMC4293246

[5] Poynter JN, Siegmund KD, Weisenberger DJ, ; Colon Cancer Family Registry Investigators. Molecular characterization of MSI-H colorectal cancer by MLHI promoter methylation, immunohistochemistry, and mismatch repair germline mutation screening. Cancer Epidemiol Biomarkers Prev. 2008;17(11):3208-3215. doi:10.1158/1055-9965.EPI-08-0512 18990764 PMC2628332

[6] Venderbosch S, Nagtegaal ID, Maughan TS, . Mismatch repair status and BRAF mutation status in metastatic colorectal cancer patients: a pooled analysis of the CAIRO, CAIRO2, COIN, and FOCUS studies. Clin Cancer Res. 2014;20(20):5322-5330. doi:10.1158/1078-0432.CCR-14-0332 25139339 PMC4201568

[7] Sinicrope FA, Shi Q, Smyrk TC, . Molecular markers identify subtypes of stage III colon cancer associated with patient outcomes. Gastroenterology. 2015;148(1):88-99. doi:10.1053/j.gastro.2014.09.041 25305506 PMC4274188

[8] Tran B, Kopetz S, Tie J, . Impact of BRAF mutation and microsatellite instability on the pattern of metastatic spread and prognosis in metastatic colorectal cancer. Cancer. 2011;117(20):4623-4632. doi:10.1002/cncr.26086 21456008 PMC4257471

[9] Sinicrope FA, Shi Q, Allegra CJ, . Association of DNA mismatch repair and mutations in BRAF and KRAS with survival after recurrence in stage III colon cancers: a secondary analysis of 2 randomized clinical trials. JAMA Oncol. 2017;3(4):472-480. doi:10.1001/jamaoncol.2016.5469 28006055 PMC5498991

[10] Le DT, Durham JN, Smith KN, . Mismatch repair deficiency predicts response of solid tumors to PD-1 blockade. Science. 2017;357(6349):409-413. doi:10.1126/science.aan6733 28596308 PMC5576142

[11] Le DT, Kim TW, Van Cutsem E, . Phase II open-label study of pembrolizumab in treatment-refractory, microsatellite instability-high/mismatch repair-deficient metastatic colorectal cancer: KEYNOTE-164. J Clin Oncol. 2020;38(1):11-19. doi:10.1200/JCO.19.02107 31725351 PMC7031958

[12] Le DT, Uram JN, Wang H, . PD-1 blockade in tumors with mismatch-repair deficiency. N Engl J Med. 2015;372(26):2509-2520. doi:10.1056/NEJMoa1500596 26028255 PMC4481136

[13] Overman MJ, McDermott R, Leach JL, . Nivolumab in patients with metastatic DNA mismatch repair-deficient or microsatellite instability-high colorectal cancer (CheckMate 142): an open-label, multicentre, phase 2 study. Lancet Oncol. 2017;18(9):1182-1191. doi:10.1016/S1470-2045(17)30422-9 28734759 PMC6207072

[14] André T, Shiu KK, Kim TW, ; KEYNOTE-177 Investigators. Pembrolizumab in microsatellite-instability-high advanced colorectal cancer. N Engl J Med. 2020;383(23):2207-2218. doi:10.1056/NEJMoa2017699 33264544

[15] Casak SJ, Marcus L, Fashoyin-Aje L, . FDA approval summary: pembrolizumab for the first-line treatment of patients with MSI-H/dMMR advanced unresectable or metastatic colorectal carcinoma. Clin Cancer Res. 2021;27(17):4680-4684. doi:10.1158/1078-0432.CCR-21-0557 33846198 PMC8416693

[16] Jin Z, Sinicrope FA. Mismatch repair-deficient colorectal cancer: building on checkpoint blockade. J Clin Oncol. 2022;40(24):2735-2750. doi:10.1200/JCO.21.02691 35649217 PMC9390830

[17] Aapro MS, Köhne CH, Cohen HJ, Extermann M. Never too old? age should not be a barrier to enrollment in cancer clinical trials. Oncologist. 2005;10(3):198-204. doi:10.1634/theoncologist.10-3-198 15793223

[18] World Health Organization. Ageing and health. Accessed September 20, 2022. https://www.who.int/news-room/fact-sheets/detail/ageing-and-health

[19] Elting LS, Cooksley C, Bekele BN, . Generalizability of cancer clinical trial results: prognostic differences between participants and nonparticipants. Cancer. 2006;106(11):2452-2458. doi:10.1002/cncr.21907 16639738

[20] Biganzoli L, Aapro M. Adjuvant chemotherapy in the elderly. Ann Oncol. 2003;14(suppl 3):iii1-iii3. doi:10.1093/annonc/mdg740 12821531

[21] Surbone A, Kagawa-Singer M, Terret C, Baider L. The illness trajectory of elderly cancer patients across cultures: SIOG position paper. Ann Oncol. 2007;18(4):633-638. doi:10.1093/annonc/mdl178 17028242

[22] Papamichael D, Audisio R, Horiot JC, ; SIOG. Treatment of the elderly colorectal cancer patient: SIOG expert recommendations. Ann Oncol. 2009;20(1):5-16. doi:10.1093/annonc/mdn532 18922882

[23] Oken MM, Creech RH, Tormey DC, . Toxicity and response criteria of the Eastern Cooperative Oncology Group. Am J Clin Oncol. 1982;5(6):649-655. doi:10.1097/00000421-198212000-00014 7165009

[24] Domingo E, Niessen RC, Oliveira C, . BRAF-V600E is not involved in the colorectal tumorigenesis of HNPCC in patients with functional MLH1 and MSH2 genes. Oncogene. 2005;24(24):3995-3998. doi:10.1038/sj.onc.1208569 15782118

[25] Parsons MT, Buchanan DD, Thompson B, Young JP, Spurdle AB. Correlation of tumour BRAF mutations and MLH1 methylation with germline mismatch repair (MMR) gene mutation status: a literature review assessing utility of tumour features for MMR variant classification. J Med Genet. 2012;49(3):151-157. doi:10.1136/jmedgenet-2011-100714 22368298

[26] Cox DR. Regression models and life-tables. J R Stat Soc B. 1972;34(2):187-220.

[27] Diaz LA Jr, Shiu KK, Kim TW, ; KEYNOTE-177 Investigators. Pembrolizumab versus chemotherapy for microsatellite instability-high or mismatch repair-deficient metastatic colorectal cancer (KEYNOTE-177): final analysis of a randomised, open-label, phase 3 study. Lancet Oncol. 2022;23(5):659-670. doi:10.1016/S1470-2045(22)00197-8 35427471 PMC9533375

[28] Andre T, Shiu K-K, Kim TW, . Final overall survival for the phase III KN177 study: pembrolizumab versus chemotherapy in microsatellite instability-high/mismatch repair deficient (MSI-H/dMMR) metastatic colorectal cancer (mCRC). J Clin Oncol. 2021;39(15 suppl):3500. doi:10.1200/JCO.2021.39.15_suppl.3500

[29] Herbst RS, Baas P, Kim DW, . Pembrolizumab versus docetaxel for previously treated, PD-L1-positive, advanced non-small-cell lung cancer (KEYNOTE-010): a randomised controlled trial. Lancet. 2016;387(10027):1540-1550. doi:10.1016/S0140-6736(15)01281-7 26712084

[30] Ribas A, Kefford R, Marshall MA, . Phase III randomized clinical trial comparing tremelimumab with standard-of-care chemotherapy in patients with advanced melanoma. J Clin Oncol. 2013;31(5):616-622. doi:10.1200/JCO.2012.44.6112 23295794 PMC4878048

[31] Kang YK, Boku N, Satoh T, . Nivolumab in patients with advanced gastric or gastro-oesophageal junction cancer refractory to, or intolerant of, at least two previous chemotherapy regimens (ONO-4538-12, ATTRACTION-2): a randomised, double-blind, placebo-controlled, phase 3 trial. Lancet. 2017;390(10111):2461-2471. doi:10.1016/S0140-6736(17)31827-5 28993052

[32] Motzer RJ, Escudier B, McDermott DF, ; CheckMate 025 Investigators. Nivolumab versus everolimus in advanced renal-cell carcinoma. N Engl J Med. 2015;373(19):1803-1813. doi:10.1056/NEJMoa1510665 26406148 PMC5719487

[33] Wu Q, Wang Q, Tang X, . Correlation between patients’ age and cancer immunotherapy efficacy. Oncoimmunology. 2019;8(4):e1568810. 30906662 10.1080/2162402X.2019.1568810 PMC6422380

[34] Kugel CH III, Douglass SM, Webster MR, . Age correlates with response to anti-PD1, reflecting age-related differences in intratumoral effector and regulatory T-cell populations. Clin Cancer Res. 2018;24(21):5347-5356. doi:10.1158/1078-0432.CCR-18-1116 29898988 PMC6324578

[35] Franko J, Shi Q, Meyers JP, ; Analysis and Research in Cancers of the Digestive System (ARCAD) Group. Prognosis of patients with peritoneal metastatic colorectal cancer given systemic therapy: an analysis of individual patient data from prospective randomised trials from the Analysis and Research in Cancers of the Digestive System (ARCAD) database. Lancet Oncol. 2016;17(12):1709-1719. doi:10.1016/S1470-2045(16)30500-9 27743922

[36] Wang C, Sandhu J, Ouyang C, Ye J, Lee PP, Fakih M. Clinical response to immunotherapy targeting programmed cell death receptor 1/programmed cell death ligand 1 in patients with treatment-resistant microsatellite stable colorectal cancer with and without liver metastases. JAMA Netw Open. 2021;4(8):e2118416. doi:10.1001/jamanetworkopen.2021.18416 34369992 PMC8353537

[37] Kim RD, Kovari BP, Martinez M, . A phase I/Ib study of regorafenib and nivolumab in mismatch repair proficient advanced refractory colorectal cancer. Eur J Cancer. 2022;169:93-102. doi:10.1016/j.ejca.2022.03.026 35526308

[38] Fukuoka S, Hara H, Takahashi N, . Regorafenib plus nivolumab in patients with advanced gastric or colorectal cancer: an open-label, dose-escalation, and dose-expansion phase Ib trial (REGONIVO, EPOC1603). J Clin Oncol. 2020;38(18):2053-2061. doi:10.1200/JCO.19.03296 32343640

[39] Yu J, Green MD, Li S, . Liver metastasis restrains immunotherapy efficacy via macrophage-mediated T cell elimination. Nat Med. 2021;27(1):152-164. doi:10.1038/s41591-020-1131-x 33398162 PMC8095049

[40] Li F, Tian Z. The liver works as a school to educate regulatory immune cells. Cell Mol Immunol. 2013;10(4):292-302. doi:10.1038/cmi.2013.7 23604044 PMC4003213

[41] Lee JC, Mehdizadeh S, Smith J, . Regulatory T cell control of systemic immunity and immunotherapy response in liver metastasis. Sci Immunol. 2020;5(52):eaba0759. doi:10.1126/sciimmunol.aba0759 33008914 PMC7755924

[42] Cohen R, Buhard O, Cervera P, . Clinical and molecular characterisation of hereditary and sporadic metastatic colorectal cancers harbouring microsatellite instability/DNA mismatch repair deficiency. Eur J Cancer. 2017;86:266-274. doi:10.1016/j.ejca.2017.09.022 29055842

[43] Lenz H-J, Van Cutsem E, Luisa Limon M, . First-line nivolumab plus low-dose ipilimumab for microsatellite instability-high/mismatch repair-deficient metastatic colorectal cancer: the phase II CheckMate 142 study. J Clin Oncol. 2022;40(2):161-170. doi:10.1200/JCO.21.01015 34637336

[44] Overman MJ, Lonardi S, Wong KYM, . Durable clinical benefit with nivolumab plus ipilimumab in DNA mismatch repair-deficient/microsatellite instability-high metastatic colorectal cancer. J Clin Oncol. 2018;36(8):773-779. doi:10.1200/JCO.2017.76.9901 29355075

