# Supplementary material for: Association Between Survival and Metastatic Site in Mismatch Repair–Deficient Metastatic Colorectal Cancer Treated With First-line Pembrolizumab
Source: JAMA Netw Open. 2023 Feb 22;6(2):e230400. doi: 10.1001/jamanetworkopen.2023.0400 (PMC9947726; doi:10.1001/jamanetworkopen.2023.0400)
Supplement: Supplement. — Data Sharing Statement [file jamanetwopen-e230400-s001.pdf]

Data Sharing Statement

Saberzadeh-Ardestani. Association Between Survival and Metastatic Site in Mismatch Repair-Deficient Metastatic Colorectal Cancer Treated With First-line Pembrolizumab. *JAMA Netw Open*. Published February 22, 2023. doi:10.1001/jamanetworkopen.2023.0400

Data
Data available: Yes
Data types: Deidentified participant data
How to access data: Sinicrope.frank@mayo.edu
When available: With publication

Supporting Documents
Document types: None

Additional Information
Who can access the data: researchers whose proposed use of the data has been approved
Types of analyses: Upon reasonable request
Mechanisms of data availability: signed data access agreement
